# The utility of the *Attitudes Toward Mathematics Inventory*—*Short Form for Children* for assessing attitudes toward mathematics in primary school children

**DOI:** 10.3389/fpsyg.2026.1659707

**Published:** 2026-01-22

**Authors:** Laura Di Leonardo, Maria Anna Donati, Kimmo Vehkalahti, Caterina Primi

**Affiliations:** 1 Department of Neuroscience, Psychology, Drug Research, and Child Health (NEUROFARBA), University of Florence, Florence, Italy; 2 Center for Social Data Science, University of Helsinki, Helsinki, Finland

**Keywords:** attitudes toward mathematics, expectancy–value theory, ATMI-SF-C, primary school children, gender invariance

## Abstract

According to the Expectancy-Value Theory, attitudes toward mathematics consist of a combination of expectancy of success and task values, including intrinsic, utility, and attainment value, along with associated costs. To analyze this construct in children, understand its relationship with other constructs, and design targeted interventions, appropriate measurement is essential. This study examined the suitability of the *Attitudes Toward Mathematics Inventory*—*Short Form* (ATMI-SF), a brief measure that allows for the assessment of the key components of attitudes as defined by the Expectancy-Value Theory, in primary school children. The sample consisted of 798 Italian children (50.5% female; *M_age_* = 9.01, *SD* = 0.91). The four-factor structure was confirmed, with evidence of gender invariance and good internal consistency. Gender differences and trends in attitudes were explored. The relationships between attitudes, math anxiety, and mathematical competence were examined, highlighting their reciprocal nature. Targeted educational strategies were discussed.

## 1 Introduction

Attitudes toward mathematics encompass an individual’s evaluation of mathematical concepts and tasks along a continuum from favorable to unfavorable (Di Martino and Zan, 2010; Hannula et al., 2016; Harun et al., 2021; Kiwanuka et al., 2022; Segarra and Julià, 2021). Since attitudes toward mathematics can influence students’ behavior and academic performance, they have been extensively studied across both Western and non-Western contexts (Adelson and McCoach, 2010; Cvencek et al., 2020; Dowker et al., 2019; Hwang and Son, 2021; Khun-Inkeeree et al., 2017; Kung and Lee, 2016; Martin et al., 2020; Soni and Kumari, 2017; Uchida and Mori, 2018), and multiple theoretical frameworks have been developed to better understand their nature. Among these, one of the most empirically supported frameworks is the Expectancy-Value Theory (EVT; Eccles and Wigfield, 2020), which clearly distinguishes between cognitive components (academic self-concept) and affective components (value and enjoyment) of academic motivation. Alongside the EVT, Di Martino and Zan (2010) proposed a multidimensional model of attitudes toward mathematics comprising three closely interrelated dimensions: Emotional disposition, vision of mathematics, and perceived competence. The emotional disposition dimension reflects students’ positive or negative feelings toward engaging with mathematics. The vision of mathematics dimension involves their perception of its nature and purpose, from rigid rules to a creative problem-solving tool. The perceived competence dimension encompasses students’ beliefs about their mathematical abilities, including confidence in understanding concepts, solving problems, and achieving desired outcomes in mathematical tasks. Similarly, researchers from the Trends in International Mathematics and Science Study (TIMSS) identified enjoyment, perceived value, and confidence as core components of math attitudes (Mullis et al., 2020).

As attitudes are acquired from direct experiences or social interactions (Aiken, 1996; Triandis, 1971), children begin to develop their attitudes toward mathematics from their early experiences at school and in social settings (Aiken, 1972; Arslan et al., 2014; Capps and Cox, 1969; Gunderson et al., 2012; Hildebrand et al., 2023; Mata et al., 2012; Peixoto et al., 2024; Pepin, 2011; Perry, 2011; Soni and Kumari, 2017; Wigfield and Eccles, 2000). This perspective, in which social and cultural influences play a crucial role in the developmental trajectory of motivation, has been recently emphasized by the transition from the Expectancy-Value Model to the Situated Expectancy-Value Model (SEVT; Eccles and Wigfield, 2020).

Once acquired, attitudes can influence individual behavioral responses and are significantly related to motivation and educational outcomes (Adelson and McCoach, 2010; Chouinard et al., 2007; Cvencek et al., 2020; Dowker et al., 2019; Guo et al., 2015; Hannula et al., 2016; Wigfield et al., 2016). Students who hold favorable attitudes toward mathematics tend to engage more actively in classroom activities and demonstrate greater persistence in overcoming challenges compared to those with less favorable attitudes toward mathematics (e.g., Chen et al., 2018; Kiwanuka et al., 2022; Mullis et al., 2020), which often translate into improved mathematical performance (Cho and Hwang, 2019; Chouinard et al., 2007; Guo et al., 2015). While the relationship between attitudes toward mathematics and mathematical competence has been well documented among middle and high school students, some studies have also identified a moderate and positive association between these constructs in primary school students (Dowker et al., 2019; Else-Quest et al., 2010; Ganley and Lubienski, 2016; Harun et al., 2021; Petersen and Hyde, 2017). Early mathematical attitudes have been recognized as strong and longitudinal predictors of later achievements and academic choices (Cvencek et al., 2021), even when accounting for other cognitive-affective factors such as IQ, working memory, anxiety, and general attitudes (Chen et al., 2018). Moreover, this relationship tends to strengthen as students progress through their academic careers (Wigfield and Cambria, 2010).

The EVT (Eccles and Wigfield, 2020; Wigfield and Eccles, 2000; Wigfield et al., 2016) offers a theoretical framework to explain this phenomenon. According to this theory, academic achievement is influenced by students’ expectancy of success (i.e., individuals’ beliefs about their ability to complete tasks) and subjective task values (e.g., individuals’ interest in a given subject domain and the value they assign to this domain; Wigfield and Cambria, 2010; Wigfield et al., 2015). Expectancy of success aligns with constructs such as academic self-concept (e.g., “I am good at mathematics”) and self-efficacy (e.g., “I am confident in solving mathematical problems”), while task values include intrinsic value (i.e., the expected enjoyment one anticipates from performing the task and the actual enjoyment experienced while performing the task), utility value (i.e., the degree to which a particular task aligns with an individual’s present or future plans), attainment value (i.e., the extent to which a task provides opportunities to express and affirm important aspects of one’s core identity), and the associated costs (i.e., the perception of the effort required for a task, the impact of this effort on other important activities, and the emotional or psychological cost of pursuing it). According to EVT, students who maintain high expectations of success and perceive mathematics as valuable tend to invest substantial time and effort in studying the subject, resulting in higher mathematical competence (Chen et al., 2018; Chouinard et al., 2007; Guo et al., 2015; Hwang and Son, 2021; Mullis et al., 2020). This framework supports the hypothesis that favorable attitudes promote engagement and motivation in mathematics-related activities, contributing to improved mathematical outcomes (Berger et al., 2020; Fong et al., 2021; Gjicali and Lipnevich, 2021). The Early Math Achievement-Attitude model (Levine and Pantoja, 2021) further elucidates the developmental interplay between mathematics achievement and attitudes in young children. This model proposes that in early childhood, mathematical competence influences attitudes; however, as children grow older, this relationship becomes bidirectional, with attitudes also playing a role in shaping mathematical performance.

Students’ enjoyment, confidence, and perceived value of mathematics are closely interconnected, with changes in one component often influencing the others (Cho and Hwang, 2019). However, these relationships may vary depending on factors such as regional culture and the specific facets of attitudes being examined (Arens et al., 2019; Lee and Seo, 2021). Moreover, individual components of attitudes toward mathematics may have distinct effects on academic performance and long-term educational choices. The literature suggests that self-concepts primarily influence immediate outcomes (e.g., actual test performance; Abed and Alkhateeb, 2000; Pinxten et al., 2014; Rosenzweig et al., 2022), whereas other attitudes are more strongly related to long-term educational trajectories (e.g., course enrollment and future aspirations; Arens et al., 2011; Rosenzweig et al., 2022; Wang, 2012). In a longitudinal study, Wang (2012) found task values to be stronger predictors of students’ engagement and career choices, whereas self-concept and expectations of success were more closely associated with academic achievement.

Following these premises, the issue of measuring attitudes toward mathematics in children becomes crucial. The availability of a reliable instrument for assessing mathematics attitudes in primary school children would facilitate research into the early development of these attitudes, clarify their relationship with performance, and support the evaluation of interventions aimed at preventing the emergence of unfavorable attitudes toward mathematics (Dowker et al., 2012).

### 1.1 The assessment of attitudes toward mathematics in primary school children

Mathematical attitudes can be measured in various ways and at different levels of complexity, ranging from general emotional reactions toward mathematics to more nuanced, multidimensional models that incorporate broader beliefs and emotions about mathematics, as well as self-perceived competence (Cvencek et al., 2021). Although attitudes have been traditionally categorized as simply positive or negative (Capps and Cox, 1969; DeBellis and Goldin, 2006; Lipnevich et al., 2013), this simplistic view focuses solely on the emotional dimension of attitudes and overlooks their role in facilitating or hindering behaviors and influencing learning outcomes (Ajisuksmo and Saputri, 2017; Goldin et al., 2011; Skott, 2015). A more accurate conceptualization views attitudes toward mathematics as multidimensional, encompassing several interrelated dimensions (Davadas and Lay, 2018; Hannula, 2002; Khine and Afari, 2014). Thus, from a psychometric perspective, effectively measuring attitudes toward mathematics requires acknowledging this complexity and addressing the various components involved, including the beliefs and values that children associate with mathematics (Cvencek et al., 2021; Zan and Di Martino, 2007). Moreover, any instrument designed for use with primary school children must be both easy to administer and developmentally appropriate.

In recent years, several instruments have been developed or adapted for this age group. Some researchers have employed qualitative methods, such as drawings, written descriptions, and interviews (Quane et al., 2023), leveraging drawing as a child-friendly means to express thoughts (MacDonald, 2013). However, drawings are influenced by individual abilities (Lowenfeld and Brittain, 1964; Vygotsky, 2004), and their interpretation heavily relies on the researcher’s subjective analysis and the adequacy of the coding system, potentially leading to misinterpretations (Cheeseman and McDonough, 2019). To improve accuracy, interviews are often combine with drawings (Crawford et al., 2012). Nevertheless, this approach can be time-consuming (Quane et al., 2023). Other researchers have employed child-friendly versions of the *Implicit Association Test* (IAT), following the methodology outlined by Cvencek et al. (2011), to examine implicit associations between domains (mathematics vs. reading) and attributes such as gender (male vs. female), difficulty (e.g., ‘simple’, ‘difficult’; Hildebrand et al., 2023) or valence (e.g., ‘happy’, ‘mad’; Cvencek et al., 2021). To accommodate children’s limited attention spans, the number of critical trials is often reduced (e.g., Dunham et al., 2014; Rutland et al., 2005). However, children may exhibit greater response variability, necessitating more trials to capture the true variance of the score (Cronbach, 1951; Nunnally, 1978). Furthermore, implicit measures are generally more susceptible to various sources of error than explicit measures (e.g., momentary inattention; Buchner and Wippich, 2000; Fiedler and Bluemke, 2005; Lane et al., 2007).

Teacher observations have also been proposed as alternative indicators of students’ attitudes (Bialangi et al., 2016). However, previous research has reported mixed findings, likely due to subjective interpretations and inter-observer variability (Wen and Dubé, 2022). Brown and Abell (1965) deemed observations are inadequate for this purpose. While mixed methods could potentially mitigate some limitations of observational data, they pose several challenges, particularly in school settings. Measuring psychological constructs requires assessments that are both developmentally appropriate and feasible within the context of the study. Observational methods demand extensive observer training require an extensive training for observers and a careful management of interpretative biases. Furthermore, data collection through systematic observation requires a significant time commitment from teachers. Finally, due to the internalizing nature of attitudes, these may not be readily observable (Lane, 2003).

Self-report instruments using Likert scales (Adelson and McCoach, 2011; Dowker et al., 2019; Hacıömeroğlu, 2017; Hidayatullah and Csíkos, 2024; Prast et al., 2012) or pictorial response scales (Krinzinger et al., 2007; Massey, 2022), are the most widely used tools in research on children’s attitudes toward mathematics, being practical and cost-effective. However, some of these instruments have limitations. For instance, some focus on narrow facets such as motivation (Hidayatullah and Csíkos, 2024), or treat attitude as a unidimensional construct (Hidayatullah and Csíkos, 2024; Massey, 2022). Other instruments address multiple subdimensions but vary in length. Some of these comprise a larger number of items (Dowker et al., 2019; Krinzinger et al., 2007; Prast et al., 2012), whereas others are shorter and thus better suited for young children (Adelson and McCoach, 2011; Deieso and Fraser, 2018; Hacıömeroğlu, 2017).

Recent studies (e.g., Cvencek et al., 2021) have drawn on data from large-scale international assessments, such as the Trends in International Mathematics and Science Study (TIMSS) and the Programme for International Student Assessment (PISA), or have employed instruments developed for these surveys to assess children’s attitudes toward mathematics. These include the Students Confident in Mathematics Scale, Fourth Grade and the Students Like Learning Mathematics, Fourth Grade (Martin et al., 2016), as well as the Mathematics Self-Concept (SCMAT; Organization for Economic Co-operation and Development; OECD, 2014). TIMSS items assess both the “confidence” and “liking” dimensions of mathematical attitudes (Martin et al., 2016), but they do not cover other dimensions, such as those assessed by the Students Value Mathematics Scale, which is available for eighth-grade students but not for fourth-grade students. PISA items assess multiple dimensions of students’ beliefs about mathematics, including interest, motivation, and self-concept (OECD, 2014), although the Mathematics Self-Concept contains too few items to evaluate each dimensions separately.

### 1.2 The present study

In this study, we focused on one of the most widely used self-report instruments for assessing attitudes toward mathematics, i.e., the *Attitudes Toward Mathematics Inventory* (ATMI; Tapia and Marsh, 2004), from which two short forms have been developed (Lim and Chapman, 2013; Lin and Huang, 2014). The first shortened version, developed by Lim and Chapman (2013), consists of 19 items and was obtained by eliminating highly correlated items without compromising the instrument’s properties. Building upon this version, Lin and Huang (2014) further reduced the instrument to a 14-item version through an exploratory factor analysis (EFA) procedure. The resulting instrument has been adapted into several languages and has shown good psychometric properties, including in Italian university students (Primi et al., 2020). It has the advantage of being brief and multidimensional, reflecting the four-factor structure of the original scale (Tapia and Marsh, 2004): Value, Self-confidence, Enjoyment, and Motivation. According to Tapia and Marsh’s (2004) definition, the Value subscale pertains to students’ beliefs regarding the usefulness and value of mathematics in daily life; the Self-confidence subscale refers to confidence in self-ability to learn and perform well on math tasks; the Enjoyment subscale reflects the degree to which students enjoy mathematics; finally, the Motivation subscale assesses students’ interest in mathematics and their willingness to continue studying it. Thus, the ATMI has the advantage of being a comprehensive measure that allows for the assessment of the key components of attitudes toward mathematics as defined by the Expectancy-Value Model. The ATMI has also been validated with samples of primary school children. Hacıömeroğlu (2017a) adapted the scale to Turkish-speaking children, using Lim and Chapman’s (2013) version as a starting point. Through exploratory and confirmatory factor analysis procedures, the author developed a 17-item scale with three factors combining Enjoyment and Motivation into a single dimension. Recently, Feng et al. (2024) adapted the 14-item version of the ATMI by Lin and Huang (2014) to Chinese primary school students, confirming the four-factor structure of the original scale.

The present study aimed to evaluate the psychometric properties of the *Attitudes Toward Mathematics Inventory*—*Short Form* (ATMI-SF; Lin and Huang, 2014) in primary school children, keeping the Expectancy-Value Theory in mind, especially for the validity purposes. Our general goal was to provide researchers and educators with a version of the instrument specifically designed for younger children, the *Attitudes Toward Mathematics Inventory*—*Short Form for Children* (ATMI-SF-C). We focused on children attending the 3rd, 4th, and 5th grades of primary school, based on the hypothesis that, by the 3rd grade, children would have accumulated sufficient experiences with mathematics to develop formed attitudes toward the subject (Eccles et al., 1993). Additionally, compared to their counterparts in the earlier grades, they were expected to have acquired sufficient reading and comprehension skills to independently complete a self-report instrument. To ensure developmental appropriateness, the scale was linguistically adjusted to align with the vocabulary and comprehension levels typically exhibited by children aged 8–11 years. This adaptation involved simplifying complex terms, rephrasing abstract concepts into more concrete and familiar language, and ensuring that sentence structures were clear and age appropriate. Moreover, the original five-point Likert response scale was replaced with a pictorial scale consisting of five boxes with progressively increasing content to assist children in indicating their level of agreement with the statements. This adjustment aimed at enhancing accessibility and engagement by providing a more intuitive and visually guided response format. Pilot testing was conducted with a small sample of children (*n* = 10) within the target age range to assess comprehension and identify ambiguities, which led to refinements for improving clarity and accessibility.

Specifically, we aimed to investigate the dimensional structure of the ATMI-SF in children through Confirmatory Factor Analysis (CFA). Previous research by Hacıömeroğlu (2017a) identified a three-factor structure for Lim and Chapman’s (2013) scale in a sample of primary school children. Therefore, our investigation aimed to evaluate both the original four-factor model and the alternative three-factor model proposed by Hacıömeroğlu (2017a), in which the Enjoyment and Motivation subscales are combined into a single dimension, to determine the most suitable factorial structure for this age group. Additionally, we aimed to assess the measurement invariance of the best-fitting model across genders, expecting the scale to maintain the same functioning in both male and female children. Despite the extensive literature on gender differences in attitudes toward mathematics, the issue of measurement invariance between males and females remains under-explored (Atılgan and Deniz, 2023) and evidence on measurement invariance in child populations is particularly scarce, except for studies on preschool children (Arens et al., 2016). Measurement invariance is essential to ensure trait scores are comparable and hold the same meaning across groups (Reise et al., 1993). Without verifying that a measure assesses the same trait across different groups, comparisons among these groups remain ambiguous (Meade and Lautenschlager, 2004). Establishing measurement invariance will allow a fairer analysis of gender differences in children’s math attitudes.

Furthermore, we tested the reliability of the scale using McDonald’s *ω* (McDonald, 1999). Based on previous studies, we expected that, despite its brevity, the scale would exhibit a good internal consistency. Concerning validity, once measurement invariance was demonstrated, the study aimed to assess gender differences in mathematical attitudes. The literature broadly supports that males hold more favorable attitudes toward mathematics than females from primary school (Cvencek et al., 2014; Cvencek et al., 2021; Else-Quest et al., 2010; Ganley and Lubienski, 2016; Gunderson et al., 2018) through high school (Berger et al., 2020; Else-Quest et al., 2010; Else-Quest et al., 2013; Jiang et al., 2024; Rodríguez et al., 2020). Studies, both longitudinal and cross-sectional, conducted in diverse cultural contexts, demonstrated that males typically exhibit higher self-confidence, greater enjoyment, and lower anxiety regarding mathematics than females (Chen et al., 2023; Else-Quest et al., 2010; Feng et al., 2024; Fredricks and Eccles, 2002; Ganley and Lubienski, 2016). Males generally perceive themselves as more competent than females (Jiang et al., 2024; Rodríguez et al., 2020) and tend to attribute their academic success to ability while attributing poor performance to a lack of effort (Beibei et al., 2022; Xie and Liu, 2023). These differences are shaped by environmental factors (Wang et al., 2023; Zander et al., 2020), particularly by exposure to gender stereotypes portraying girls as less capable and talented (Ceci, 2017; Cvencek et al., 2021; Del Río et al., 2020; Feng et al., 2024; Lazarides et al., 2019; Muzzatti and Agnoli, 2007; Napp and Breda, 2022). Both the 2022 PISA report (OECD, 2023) and the studies by Anokye-Poku and Ampadu (2020) and Bashir et al. (2023) emphasize gender-based differences in both performance and motivation, with females’ higher anxiety serving as a potential contributing factor to these disparities. Tsai et al. (2018), using 2012 PISA data, found that the gender gap in mathematics performance, which favors boys in Western countries, does not hold in some East Asian countries such as Japan and South Korea. However, while gender differences in mathematical self-concept appear to be consistent in most countries, even after accounting for mathematical achievement (e.g., Mejía-Rodríguez et al., 2021), findings regarding gender differences in enjoyment and perceived value of mathematics are less consistent (Dowker et al., 2019). For instance, Dowker et al. (2012) discovered that primary school boys rated their mathematical abilities higher than girls, even though there were no significant differences between genders in other attitudes or actual performance. Likewise, Ganley and Lubienski (2016) found that while primary school children exhibited minimal gender differences in their enjoyment and interest in mathematics, boys demonstrated higher levels of confidence. Consistent with prior research, we hypothesized that male children would exhibit more favorable attitudes toward mathematics. Specifically, while we expected female children to acknowledge the importance and value of mathematics as male children, we anticipated they would likely exhibit a less positive self-concept in mathematics compared to their male counterparts.

The study also aimed to investigate trends in both the total score and subscale scores of the attitude toward mathematics from the 3rd to the 5th grade, separately by gender. According to the literature, social and cultural factors significantly influence the developmental trajectory of motivation (Eccles and Wigfield, 2020), with gender stereotypes playing a key role. Research has shown significant differences in attitudes toward mathematics between males and females, with boys often expressing more favorable attitudes (Cvencek et al., 2014; Cvencek et al., 2021; Else-Quest et al., 2010; Ganley and Lubienski, 2016; Gunderson et al., 2018). Based on the existing literature, we aimed to explore how attitudes toward mathematics develop across grades and whether these trends differ by gender.

Moreover, we investigated the relationships between the ATMI-SF-C subscale scores and theoretically related constructs, such as mathematical competence and math anxiety. Although some authors conceptualize mathematics anxiety as a component of mathematical attitude (e.g., Casey and Ganley, 2021; Dowker et al., 2019), characterized by feelings of tension and apprehension that interfere with numerical manipulation and problem-solving (Richardson and Suinn, 1972), other researchers (e.g., Cvencek et al., 2021) distinguish between the two constructs. Cvencek et al. (2021) argue that an individual may hold a negative attitude toward mathematics without necessarily experiencing mathematics anxiety. Math anxiety is generally associated with more severe and sometimes uncontrollable emotional responses in situations involving numerical activities, heightened tension in testing contexts, and autonomic reactions. DeBellis and Goldin (2006) distinguish between emotions, which are transient affective states, and attitudes, which are moderately stable predispositions integrating affective and cognitive components. We align with this second perspective, arguing that mathematics anxiety should not be conceptualized as an attitude due to its predominantly emotional rather than evaluative nature. Although extensively studied, the relationship between mathematical attitudes and math anxiety remains unclear (Levine and Pantoja, 2021). Some authors suggest that specific mathematical attitudes serve as foundational to others, functioning as “hub” attitudes that shape and influence the broader network of mathematical beliefs and emotions, including math anxiety (Casey and Ganley, 2021; Feng et al., 2024; Levine and Pantoja, 2021). However, these relationships may vary due to participants’ age and cultural factors. For instance, research conducted in Eastern countries suggests that enjoyment has a stronger influence on later self-confidence (Chen et al., 2023; Cvencek et al., 2021; Feng et al., 2024), whereas findings from Western countries indicate the opposite path (Arens et al., 2019).

Debate also persists regarding the relationship between math attitude and performance, as research on young children has yielded mixed findings. Some authors have found weak o non-significant associations between attitudes toward mathematics and mathematical competence in this age group (Cain-Caston, 1993; Krinzinger et al., 2009). However, as children progress through the upper primary school grades, they experience a series of successes and failures that may influence their attitudes and self-confidence in mathematics (Eccles et al., 1993). Accordingly, the effects of attitude on performance may become more evident in late primary school. The relationship between attitude toward mathematics, mathematics anxiety, and mathematical competence was therefore explored in studying the validity of the ATMI-SF-C. We expected to find a positive correlation between each dimension of the ATMI-SF-C and mathematical competence (Ma and Kishor, 1997) and negative correlations between ATMI-SF-C dimensions and math anxiety (Ashcraft and Krause, 2007; Hembree, 1990). Specifically, we expected a stronger correlation between the Self-Confidence subscale and math anxiety, given that this subscale is composed of items originally derived from separate subscales measuring confidence and math anxiety (Tapia, 1996). Moreover, the present study aimed to deepen the current understanding of the mechanisms underlying the relationship between attitudes toward mathematics and mathematical competence. Indeed, the underlying developmental mechanisms through which attitudes toward mathematics influence mathematical development, particularly in young children, remain poorly understood. Casanova et al. (2021) identified math anxiety as one such mechanism. From the perspective of Control-Value Theory, which integrates assumptions from Expectancy-Value Theory, mathematics attitudes are likely to influence math competence through emotions that shape motivation and behaviors leading to changes in mathematical competence (Pekrun, 2006; Pekrun et al., 2007; Pekrun and Perry, 2014). In this framework, self-perceived mathematical competence (e.g., confidence in solving mathematical problems in class or in a test) and the subjective value attributed to mathematics (e.g., the value of learning mathematics) represent two key motivational antecedents of math anxiety (Li et al., 2021; Pekrun, 2021; Pekrun et al., 2023; Ramirez et al., 2018). Negative emotions such as math anxiety can, in turn, reduce motivation and lead to task avoidance, potentially hindering mathematical competence (Pekrun, 2006). Thus, to explore the relationship between attitudes toward mathematics and mathematical competence, we tested a mediation model that examined the effects of the different facets of attitude toward mathematics (independent variables) on mathematical competence (dependent variable) through math anxiety (mediator). Considering that existing literature has highlighted variations in attitudes toward mathematics based on gender (Dowker et al., 2012), we also included this variable as a covariate in the mediation model to account for its effect. Building on existing evidence, we anticipated that children with more favorable attitudes toward mathematics would exhibit lower math anxiety (Ashcraft and Krause, 2007) and perform better in math tasks (Dowker et al., 2019). Furthermore, considering prior studies linking higher math anxiety to reduced mathematical competence (Carey et al., 2016; Commodari and La Rosa, 2021; Zhang et al., 2019), and evidence suggesting that this relationship seems to become stronger starting from third grade (Cargnelutti et al., 2017; Lauer et al., 2018), we expected that children with more favorable attitudes toward mathematics would experience lower math anxiety, which, in turn, would enable them to perform better in mathematical tasks. Concerning the role of the different facets of math attitude, studies have yielded conflicting results regarding the identification of the attitudes that serve as the strongest predictors of academic performance. However, there is evidence suggesting that individual components of attitudes toward mathematics may have distinct effects on academic performance, with self-confidence potentially exerting the most significant role (Abed and Alkhateeb, 2000; Pinxten et al., 2014).

Consistent with prior literature highlighting the recursive nature of the relationship between these constructs, the present study also explored the reverse direction of the association between mathematical competence and attitudes toward mathematics, considering math anxiety as a potential mediating mechanism. According to the Early Math Achievement-Attitude Model (Levine and Pantoja, 2021), early mathematical competence can shape children’s developing attitudes toward mathematics, particularly in the primary school years. Specifically, children who experience success in mathematical tasks are more likely to develop a positive self-concept as learners of mathematics, perceive mathematics as valuable, and show greater enjoyment and persistence in mathematical activities. Conversely, repeated difficulties or poor performance may contribute to the emergence of math anxiety, which, in turn, may give rise to unfavorable attitudes toward mathematics (Levine and Pantoja, 2021). Accordingly, in the present study, we also tested a mediation model in which mathematical competence (independent variable) was hypothesized to be related to the different facets of attitude toward mathematics (dependent variables) through math anxiety (mediator). Gender was also included as a covariate, given consistent evidence of gender-related differences in both mathematical competence and affective-motivational variables related to mathematics (Dowker et al., 2012). Based on previous literature, we expected that higher mathematical competence would be associated with lower math anxiety, which, in turn, would be related to more favorable attitudes toward mathematics.

## 2 Materials and methods

### 2.1 Participants

A total of 798 children (50.5% female; *M_age_* = 9.01, *SD* = 0.91) were recruited from three primary schools in central Italy. Specifically, 33.2% of the participants attended the 3rd grade, 29.7% the 4th grade, and 38.1% the 5th grade.

To recruit participants, the project was presented to a large selection of public and private primary schools in Tuscany. Three schools, located in two different provinces and belonging to urban and suburban areas, which can be considered representative of the entire regional territory, agreed to participate.

Italian schools follow the National Guidelines for the Curriculum of Early Childhood Education and the First Cycle of Education (Ministerial Decree No. 254, November 16, 2012). In the context of arithmetic skill development, primary school students progressively enhance their ability to perform basic operations (addition, subtraction, multiplication, and division) using natural numbers, decimals, and integers. Early education focuses on mental calculation and simple operations, while more complex calculations, particularly those involving decimals, are introduced in the later grades. Problem-solving and real-world applications are considered central components in mathematics learning.

The study’s objectives and methodology were approved by the collegiate bodies of each school. All phases of the research project were developed and implemented according to the code of ethics of the Italian Association of Psychology (2015, revised 2022), which draws inspiration from the Declaration of Helsinki (1964/2013). Ethical approval was obtained from the Institutional Review Board of the University of Florence (Approval No. 341/2024). Informed consent was obtained from the parents of all participants before their inclusion in the study, and confidentiality of their data was ensured throughout the research process.

### 2.2 Measures and procedure

The *Attitude Toward Mathematics Inventory*—*Short Form* (ATMI-SF; Lin and Huang, 2014; Italian version: Primi et al., 2020) is a 14-item self-report instrument designed to measure attitudes toward mathematics. The wording of the items was adapted to align with the experiences and comprehension levels of the children. To assist children in selecting the response option that most accurately reflects their experience, participants were asked to indicate their level of agreement with a series of sentences regarding mathematics using a pictorial scale consisting of five boxes with increasing content, corresponding to a Likert response scale from 1 (*I do not agree much*) to 5 (*I very much agree*). Higher scores indicated a more favorable attitude toward mathematics. The psychometric soundness of the original ATMI-SF was established by Lin and Huang (2014) through a CFA that supported a four-factor, 14-item structure (χ^2^ = 320.12, df = 71, *Goodness of Fit Index* = 0.96, *Standardized Root Mean Square Residual* = 0.035, *Root Mean Square Error of Approximation* = 0.055, *Tucker-Lewis Index* = 0.98, *Comparative Fit Index* = 0.98). All standardized factor loadings were significant (*p* < 0.001) and ranged from 0.65 to 0.84. Composite reliability coefficients ranged from 0.78 to 0.85, and average variance extracted (AVE) values ranged from 0.53 to 0.59, demonstrating satisfactory reliability and convergent validity. Discriminant validity was further supported, as the square roots of the AVE for each construct exceeded the corresponding interconstruct correlations.

The *Elementary School - Abbreviated Math Anxiety Scale* (ES-AMAS; Caviola et al., 2017) is a self-report instrument measuring math anxiety in children. The scale comprises nine items that evaluate two aspects of math anxiety: Anxiety related to learning (5 items, e.g., “*When you use the number line*”) and anxiety related to evaluation (4 items, e.g., “*When the teacher asks you to solve a math problem*”). Participants were asked to indicate the degree of anxiety experienced in various situations regarding mathematics using a pictorial scale consisting of five boxes with increasing content, corresponding to a response scale from 1 (*Little anxiety*) to 5 (*Much anxiety*). This instrument has demonstrated good reliability and validity in a sample of late primary school pupils (Caviola et al., 2017). In the original validation study by Caviola et al. (2017), the ES-AMAS demonstrated satisfactory internal consistency, with Cronbach’s *α* = 0.77 (95% CI [0.74–0.79]) for the total scale, *α* = 0.64 (95% CI [0.60–0.68]) for the Learning subscale, and *α* = 0.74 (95% CI [0.70–0.77]) for the Evaluation subscale. All item–total correlations exceeded 0.31, and no increase in reliability was observed upon item deletion. Moreover, the ES-AMAS demonstrated moderate positive correlations with general anxiety and low negative correlations with mathematical competence (Caviola et al., 2017). In the present study, internal consistency was comparable, with McDonald’s *ω* = 0.83 (95% CI [0.81–0.85]) for the total scale, *ω* = 0.76 (95% CI [0.73–0.79]) for the Learning subscale, and *ω* = 0.77 (95% CI [0.74–0.80]) for the Evaluation subscale. Item–rest correlations ranged from 0.45 to 0.61, providing further evidence of the reliability of the measure in the current sample.

The AC-MT 6–11 years (Cornoldi et al., 2012) is a standardized assessment tool for evaluating mathematical competence in Italian primary school children. It consists of both group-administered subtests (e.g., Written operations, Numerosity judgment, Numerosity ordering) and individually administered subtests (e.g., Mental calculation, Enumeration, Dictation of numbers). For this study, a selection of group-administered subtests, including ascending and descending digit sorting and basic mathematical operations, was administered. These subtests were chosen for their alignment with fundamental mathematical competencies and their relevance to the Italian primary school curriculum. The ascending digit sorting task requires participants to arrange a series of digits in ascending order, from the smallest to the largest number. Successful completion of this task assesses children’s understanding of the numerical magnitude and their ability to compare and arrange numbers. The descending digit sorting task asks children to reorder a set of digits in descending order, from the largest to the smallest number. This task evaluates children’s understanding of number relationships in the opposite direction, further assessing their numerical comprehension and cognitive organizational skills. Both the ascending digit sorting and descending digit sorting tasks consist of five items, preceded by a practice item. The basic mathematical operations subtest requires children to perform basic arithmetic operations, including addition, subtraction, multiplication, and division. It consists of eight items. The difficulty level increases as the task progresses, starting with simple single-digit calculations and advancing to more complex operations. This task measures a child’s competence in performing fundamental mathematical calculations and understanding basic arithmetic principles. The speed and accuracy with which children complete these tasks provide insight into their operational proficiency in mathematics. Each task was group-administered and was terminated when 90% of participants had completed it. For each subtest, the score was determined by counting the number of correct answers, and a total score was subsequently calculated. The AC-MT 6–11 years has demonstrated good test–retest reliability, with an average correlation coefficient of 0.64 for the group-administered subtests. Additionally, the measure showed adequate concurrent validity, as indicated by an average correlation of 0.55 between the group-administered subtests and teachers’ evaluations of students’ mathematical competence. In the present study, correlations among subtests were significant and positive (ranging from 0.26 to 0.53).

The administration was conducted collectively during school hours by a trained research psychologist team. The examiner provided instructions to the children and addressed their questions. Subsequently, children completed the paper-and-pencil protocol individually. The instruments were administered in the following sequence: ATMI-SF-C, ES-AMAS, and AC-MT 6–11 years. The entire procedure took approximately 40 min.

## 3 Results

### 3.1 Dimensionality

Firstly, item distributions and descriptives were analyzed to assess normality (Supplementary Table S1). Skewness values ranged from −1.56 to 0.34, while Kurtosis indices ranged from −1.31 to 0.2.42. However, the deviation of a few items from normality can be considered negligible (Ghasemi and Zahediasl, 2012).

To verify the fit of the four-factor model, a CFA was conducted using the maximum likelihood method. The following indicators were used: the Comparative Fit Index (CFI; Bentler, 1990), the Tucker-Lewis Index (TLI; Tucker and Lewis, 1973), and the Root Mean Square Error of Approximation (RMSEA; Steiger and Lind, 1980). CFI and TLI values above 0.90 indicate acceptable fit, and above 0.95 indicate excellent fit. RMSEA values below 0.08 are acceptable, and below 0.05 are good (Kline, 2023).

**Figure 1:**
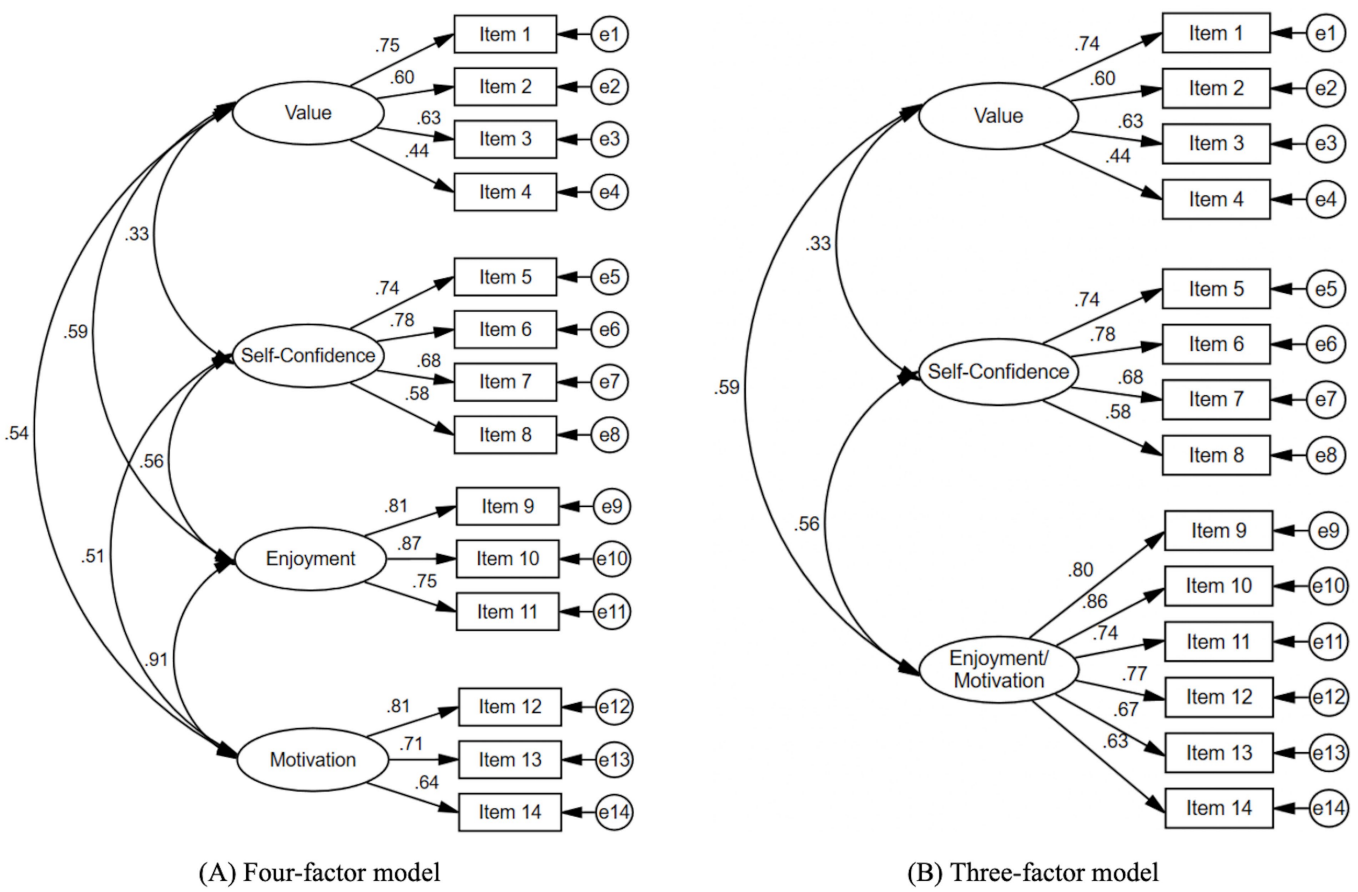
Four-factor **(A)** and three-factor **(B)** structures of the attitudes toward mathematics inventory-short form-children in primary school students. Standardized factor loadings are all significant at *p* < 0.001.

The results indicated an excellent fit for the original four-factor model (CFI = 0.973; TLI = 0.965; RMSEA = 0.045, 90% CI [0.038, 0.053]). All factor loadings exceeded 0.30, with values ranging from 0.44 to 0.87 (*p* < 0.001). The correlations between factors were significant at the 0.001 level and positive, ranging from 0.33 to 0.91 (Figure 1A).

The CFA was then replicated using a three-factor structure, where items from both *Enjoyment* and *Motivation* subscales loaded on a single factor. The results indicated a good model fit (CFI = 0.965; TLI = 0.957; RMSEA = 0.050, 90% CI [0.043,0.058]). All factor loadings were above 0.30, ranging from 0.45 to 0.86 (*p* < 0.001). The correlations between the factors were significant at the 0.001 level, ranging from 0.33 to 0.59 (Figure 1B).

To determine the most parsimonious model, we employed the Akaike Information Criterion (AIC; Akaike, 1974) and the Bayesian Information Criterion (BIC; Schwarz, 1978; Akaike, 1974) for model comparison, following the methodology outlined by Vandenberg and Grelle (2009). After comparing the 4-factor structure with the 3-factor structure using these criteria, the results indicated a lower level of information loss for the original 4-factor structure (Table 1). Thus, subsequent analyses were carried out based on the four-factor model.

**Table 1:** Goodness-of-fit statistics for the four-factor and the three-factor models of the ATMI-SF-C in primary school children.

Model	*χ* ^2^	*df*	*p*	*χ*^2^/*df*	TLI	CFI	RMSEA [90% CI]	AIC	*BIC*
Four-factor model	187.769	71	<0.001	2.645	0.965	0.973	0.045 [0.038–0.053]	255.769	414.961
Three-factor model	222.638	74	<0.001	3.009	0.957	0.965	0.050 [0.043–0.058]	284.638	429.784

*χ*^2^ = chi square test; df = degrees of freedom; CFI = comparative fit index; TLI = Tuker-Lewis index; RMSEA = Robust root mean square error of approximation; 90% CI = 90% Confidence Interval; AIC = Akaike Information Criterion; BIC = Bayesian Information Criterion. *n* = 798.

### 3.2 Invariance across gender

In the next step, a multi-group CFA was conducted to test the gender invariance (males vs. females) of the four-factor structure. Firstly, the model was tested separately in the two groups (Byrne, 2004). Subsequently, the four steps of invariance were considered (Timmons, 2010): configural invariance (Model 1), metric invariance (Model 2), strong invariance (Model 3), and strict invariance (Model 4). Changes in CFI, TLI, and RMSEA were used to test invariance; ΔCFI ≤ 0.01, ΔTLI ≤ 0.01, and ΔRMSEA ≤ 0.015 were considered evidence of invariance (Chen, 2007; Cheung and Rensvold, 2002).

To test the gender invariance of the four-factor model, a multi-group CFA was conducted using data from 393 males and 398 females. The model exhibited good fit among males (CFI = 0.963, TLI = 0.953, RMSEA = 0.050 (90% CI [0.038, 0.062])), with factor loadings ranging from 0.47 to 0.88 and being significant at the 0.001 level. Similarly, excellent fit indices were also observed among females (CFI = 0.977, TLI = 0.970, RMSEA = 0.043 (90% CI [0.030, 0.056])). The standardized factor loadings ranged from 0.41 to 0.86 and were significant at the 0.001 level. In line with the recommended practice for testing measurement invariance (Dimitrov, 2010; Little, 1997; Vandenberg and Lance, 2000), firstly, the independence model was fitted (*χ^2^* = 4330.755, *df* = 182, *p* < 0.001). The differences in CFI and RMSEA values were less than 0.01 and 0.015, respectively (Cheung and Lau, 2012), indicating full-scale invariance across gender (Table 2).

**Table 2:** Fit statistics of the ATMI-SF-C for each level of structural and measurement invariance across gender.

Model	*χ*^2^ (*df*)	*χ*^2^/*df*	*p*	CFI	RMSEA [90% CI]	Model Comparison	Δ*χ*^2^	Δ*df*	*p*	ΔCFI	ΔRMSEA
1. Invariance of model configuration (*Configural invariance*)	263.881 (143)	1.858	<0.001	0.971	0.033 [0.027–0.039]	–	–	–	–	–	–
2. Invariance of factor loadings (*Weak or Metric invariance*)	277.183 (152)	1.824	<0.001	0.970	0.032 [0.026–0.038]	Model 1–Model 2	13.302	10	0.207	0.001	0.001
3. Invariance of intercepts (*Scalar or Strict Invariance*)	330.333 (166)	1.990	<0.001	0.960	0.035 [0.030–0.041]	Model 2–Model 3	53.150	14	0.000	0.010	0.003
4. Invariance of structural variances/covariances	347.150 (176)	1.972	<0.001	0.959	0.035 [0.030–0.041]	Model 3a–Model 4	16.818	10	0.078	0.001	0.000
5. Invariance of measurement error variances/covariances	377.162 (190)	1.985	<0.001	0.955	0.035 [0.030–0.041]	Model 4–Model 5	30.012	14	0.008	0.004	0.000

*χ*^2^ = chi square test; df = degrees of freedom; CFI = robust comparative fit index; RMSEA = robust root mean square error of approximation; Δχ^2^ = Satorra-Bentler scaled difference; Δdf = difference in degrees of freedom between nested models; p = probability value of Δχ^2^ test; ΔCFI = difference between robust CFIs of nested models. *n* = 791 (Males = 393; Females = 398).

### 3.3 Reliability

Reliability was investigated in terms of internal consistency using McDonald’s *ω* coefficient. McDonald’s *ω* for the entire scale was 0.87 (95% CI [0.86, 0.89]). No increase in omega values was observed when one of the items was eliminated. McDonald’s *ω* was 0.70 (95% CI [0.67–0.74]) for the Value subscale, 0.78 (95% CI [0.77–0.81]) for the Self-Confidence subscale, and 0.85 (95% CI [0.83–0.87]) for the Enjoyment subscale, and 0.77 (95% CI [0.74–0.79]) for the Motivation subscale. According to the cut-offs proposed by the European Federation of Psychological Assessment (EFPA; Evers et al., 2013), the internal consistency values were adequate for the Value, Self-Confidence, and Enjoyment subscales and good for the Motivation subscale.

### 3.4 Validity

#### 3.4.1 Differences and trend analysis by gender

Once we verified gender invariance, we compared the mean scores of males and females on the ATMI-SF-C. The total score of the ATMI-SF-C was significantly (*t*(789) = 5.09, *p* < 0.001) higher in males (*M* = 53.15, *SD* = 10.51) compared to females (*M* = 49.31, *SD* = 10.70), with a moderate effect size (*Cohen’s d* = 0.36). Regarding the different domains of attitude toward mathematics, we found a significant difference between males and females in all the subscales except for the Value subscale (Table 3).

**Table 3:** Differences between average ATMI-SF-C scores of males and females.

Subscale/Total score	Mean (SD)		*t*	*df*	*p*	Cohen’s *d*
	Males (*n* = 393)	Females (*n* = 398)				
Value	16.09 (3.08)	15.77 (3.00)	1.46	789	0.145	0.10
Self-Confidence	15.93 (3.82)	14.75 (4.14)	4.17	789	<0.001	0.30
Enjoyment	11.60 (3.33)	10.58 (3.53)	4.17	789	<0.001	0.30
Motivation	9.53 (3.42)	8.20 (3.42)	5.47	789	<0.001	0.39
ATMI-SF-C total score	53.15 (10.51)	49.31 (10.70)	5.09	789	<0.001	0.36

*n* = 791. ATMI-SF-C = Attitudes toward mathematics inventory – short form for children.

**Figure 2:**
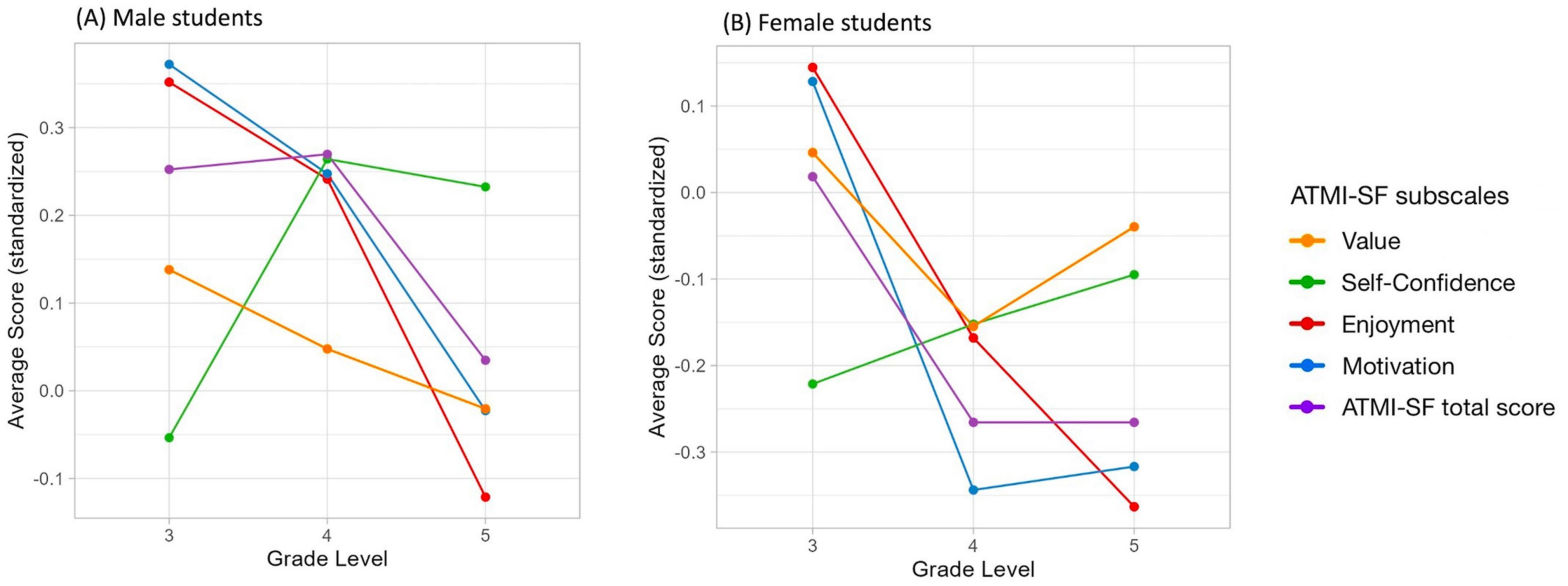
Trends in standardized total and subscale scores of the ATMI-SF-C in male **(A)** and female **(B)** students.

Then, separately by gender, we analyzed trends over school grades by considering the standardized total and subscale scores of the ATMI-SF-C (Figure 2). In both groups, attitudes toward mathematics appeared less favorable from the third to the fifth grade of primary school. When considering each component, self-confidence increased over time, while all other components tended to become less favorable. Notably, the Enjoyment and Motivation scores seemed to experience the most pronounced decrease over the years. However, this trend seemed more pronounced and emerged earlier in the female group. Additionally, in the female group, although the Self-Confidence score appeared to increase over the grades, it consistently remained lower than that of the males.

#### 3.4.2 Relationships with mathematical competence and mathematics anxiety

The total and subscale scores of the ATMI-SF-C showed significant but weak positive correlations with mathematical competence. Each dimension also correlated negatively with math anxiety. As predicted, among the subscales of the ATMI-SF-C, the Self-Confidence subscale had the highest correlation with math anxiety (Table 4).

**Table 4:** Correlations between the subscales and the total score of the ATMI-SF-C, the ES-AMAS, and the AC-MT 6–11 years.

Variable	1	2	3	4	5	6	7	8	9
1. Value	–								
2. Self-Confidence	0.237^***^	–							
3. Enjoyment	0.456^***^	0.464^***^	–						
4. Motivation	0.398^***^	0.420^***^	0.744^***^	–					
5. ATMI-SF-C	0.647^***^	0.725^***^	0.864^***^	0.832^***^	–				
6. Learning	−0.143^***^	−0.551^***^	−0.219^***^	−0.190^***^	−0.380^***^	–			
7. Evaluation	−0.212^***^	−0.617^***^	−0.362^***^	−0.369^***^	−0.529^***^	0.560^***^	–		
8. ES-AMAS	−0.201^***^	−0.661^***^	−0.328^***^	−0.316^***^	−0.514^***^	0.885^***^	0.881^***^	–	
9. AC-MT	0.117^***^	0.344^***^	0.195^***^	0.169^***^	0.278^***^	−0.363^***^	−0.282^***^	−0.365^***^	–

ATMI-SF-C = Attitudes Toward Mathematics Inventory – Short Form for Children, ES-AMAS = Elementary School – Abbreviated Math Anxiety Scale, AC-MT 6–11 years = Calculation Skills Assessment Test 6–11 years. ***p < 0.001, *n* = 798.

**Figure 3:**
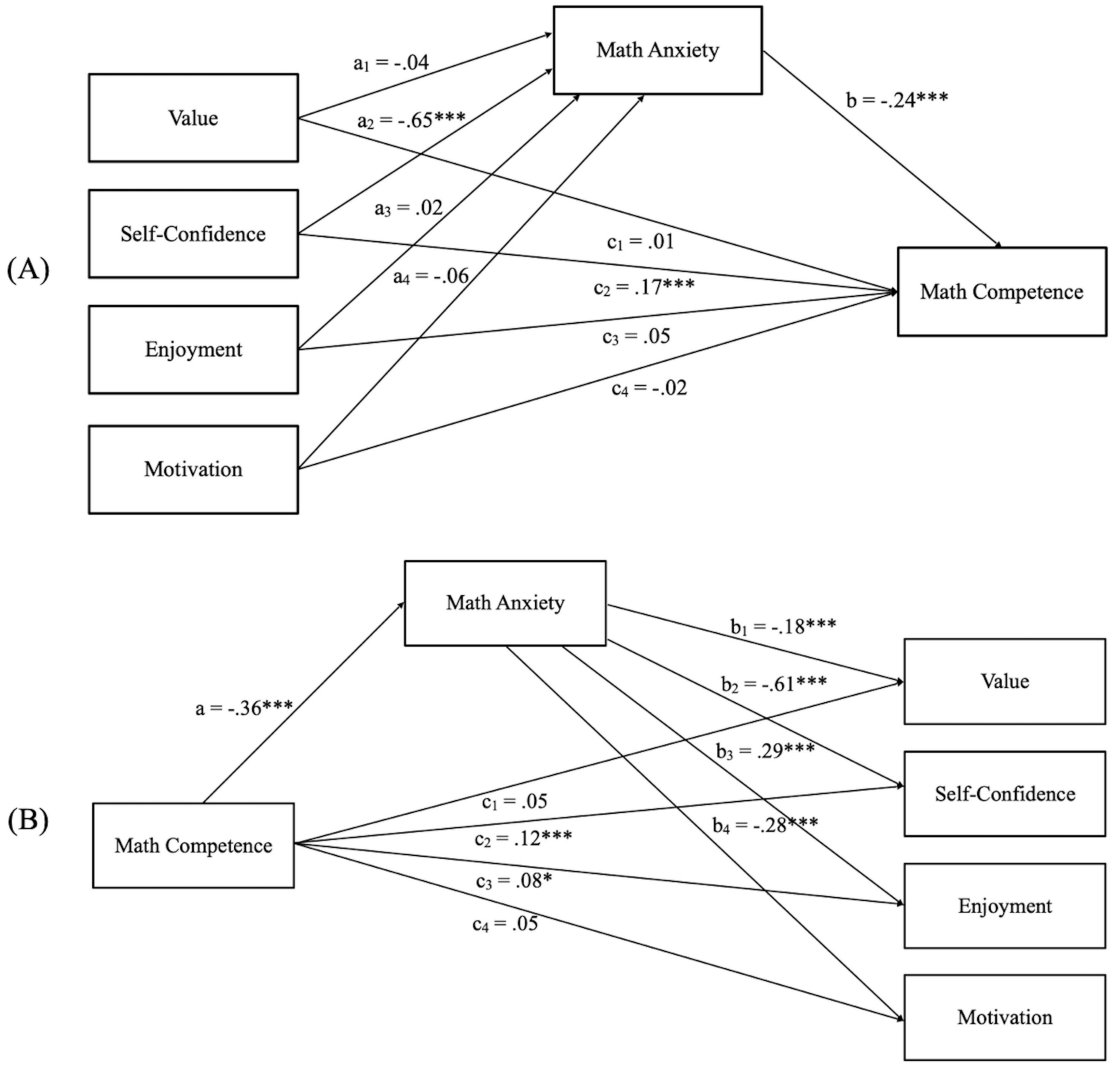
Path coefficients for the mediation analysis on math competence **(A)** and for the mediation analysis on attitudes toward mathematics **(B)**. The a, b, and c coefficients represent standardized ordinary least squares regression coefficients. ***p < 0.001, *p < 0.05.

Then, the mediation model was estimated to derive the effects of attitudes toward mathematics on math competence through math anxiety. Specifically, the indirect effect was calculated as the product of the ordinary least squares (OLS) regression coefficient estimating math anxiety from the ATMI-SF-C subscales (i.e., Paths a1, a2, a3, and a4 in Figure 3A) and the OLS regression coefficient estimating math competence from math anxiety while controlling attitudes toward mathematics (i.e., Path b in Figure 3A). A bias-corrected bootstrap 95% confidence interval (CI) for this product that does not include zero indicates a significant indirect effect (Hayes, 2009; Preacher and Hayes, 2008). Results showed a significant positive indirect effect of attitude toward mathematics on math competence through math anxiety (point estimate = 0.17, 95% CI = [0.08, 0.21]). In particular, self-confidence was the only attitude dimension that showed a significant effect on math competence, both directly and indirectly through math anxiety, is self-confidence. The proportion mediated (PM) was 0.46, indicating that 46% of the total effect of attitude toward mathematics on math competence was mediated by math anxiety. To further explore whether these effects varied by gender, we included gender as an additional explanatory variable in the mediation model. Controlling for this covariate did not substantially alter the relationships among attitudes toward mathematics, math anxiety, and math competence. Furthermore, we examined whether gender exhibited significant direct or indirect effects on math competence and found no evidence of significance (*p* = 0.518).

Finally, following the procedure used in the previous model, the reverse mediation model was estimated to examine whether math competence was associated with attitudes toward mathematics via math anxiety, with gender included as a covariate (Figure 3B). Results indicated a significant overall indirect effect of math competence on attitudes toward mathematics through math anxiety (point estimate = 0.49, 95% CI = [0.41, 0.69]). The proportion mediated (PM) was 0.63. Gender did not substantially alter the pattern of relationships between math competence, anxiety, and attitudes. However, it exhibited significant direct effects on Enjoyment (*b* = − 0.670, *p* = 0.006) and Motivation (*b* = − 0.999, *p* < 0.001). No significant gender effects were observed for Value (*p* = 0.354) or Self-Confidence (*p* = 0.130) dimensions.

## 4 Discussion

Understanding children’s attitudes toward mathematics is essential, as early experiences significantly influence their development (Aunola et al., 2013; Mohr-Schroeder et al., 2017). This study contributes to the literature by proposing a multidimensional measure grounded in the Expectancy-Value Theory (Eccles and Wigfield, 2002), which conceptualizes attitudes as a combination of expectancy of success and task values.

The research specifically examined the suitability of the ATMI-SF-C for primary school children. The results supported the scale’s original four-factor structure - Value, Self-Confidence, Enjoyment, and Motivation - within this group, confirming that the ATMI-SF-C effectively captures the multifaceted nature of attitudes toward mathematics. This multidimensional structure reflects the complexity of attitudes toward mathematics (Cvencek et al., 2021) and aligns with the Expectancy-Value Theory (Eccles and Wigfield, 2002). A tool that captures distinct yet interrelated dimensions of attitudes toward mathematics allows for the separate analysis of their components and their differential relationships with relevant variables, such as mathematical competence. Furthermore, ensuring consistency between the ATMI-SF-C and established measures for adolescents and adults facilitates longitudinal investigations, enabling the examination of how different components of attitudes toward mathematics contribute to both short- and long-term academic outcomes.

A key contribution of this study was examining the measurement invariance of the ATMI-SF-C between male and female children, an area previously unexplored (Atılgan and Deniz, 2023). Establishing measurement invariance is essential for ensuring unbiased comparisons across groups (Reise et al., 1993), thereby advancing research on gender differences in attitudes toward mathematics. The instrument also showed good reliability and validity. Regarding gender differences, our analysis revealed that, while female children recognize the value and importance of mathematics similarly to boys, they exhibit lower self-confidence, less enjoyment in learning mathematics, and reduced motivation to pursue further studies in this field. In terms of EVT components, the results indicate that girls display lower levels of self-confidence (i.e., expectancy for success), enjoyment in mathematics (i.e., intrinsic value), and motivation to continue studying math, likely due to a perceived mismatch between task characteristics and their core self-schema (i.e., attainment value). In contrast, boys and girls do not significantly differ in their perception of the importance and utility of mathematics, which corresponds to the utility value component of the EVT model. Consistent with the EVT model (Eccles and Wigfield, 2020), the relative weight and significance of specific attitude components in both short-term achievement-related decisions and long-term activity choices are influenced by social, developmental, and psychological factors. Age and maturation affect not only their weight but also the timing of the emergence and development of the cognitive processes underlying these components (Eccles and Wigfield, 2020). Furthermore, the beliefs and behaviors of socializers, their expectations regarding children’s academic achievement, and culturally prescribed social roles, such as gender roles, also play a crucial role in shaping these components (Eccles and Wigfield, 2020). Within the domain of mathematics, considerable attention has been given to the social transmission of gender stereotypes and their role in shaping attitudes toward math (Ceci, 2017; Cvencek et al., 2021; Del Río et al., 2020; Feng et al., 2024; Lazarides et al., 2019; Muzzatti and Agnoli, 2007; Napp and Breda, 2022). Our findings are consistent with prior research indicating that girls are particularly influenced by the attitudes and gender-related beliefs of significant adults. Mathematics-related attitudes are socially transmitted through various sources, including teachers (Beilock et al., 2010) and parents (Gunderson et al., 2012; Hildebrand et al., 2023; Peixoto et al., 2024). It is plausible that adults, influenced by gender stereotypes, tend to hold higher expectations for boys’ mathematical performance while conveying less favorable attitudes toward mathematics to girls (Gunderson et al., 2012; Wigfield and Eccles, 2000). This may contribute to gender differences in mathematics self-concept and in specific subject value components, particularly those more closely linked to intrinsic motivation. Regarding the perceived utility value of mathematics, which, according to the EVT model, is related to extrinsic motivation (Eccles and Wigfield, 2020; Ryan and Deci, 2020), our results align with previous studies showing no significant gender differences in how adolescents view the importance of mathematics (e.g., Jacobs et al., 2002; Watt, 2004). This suggests that, within educational contexts, the utility value of mathematics is conveyed equally to both boys and girls (Watt, 2004).

Trend analysis by gender showed that both male and female children experience a decline in favorable attitudes toward mathematics from the third to the fifth grade of primary school. Regarding individual components, self-confidence slightly increased over time, while the other components tended to become less favorable. Notably, the Enjoyment and Motivation scores showed the most pronounced decline over the years. Furthermore, this decline appeared more pronounced and occurred earlier in the female group. Additionally, despite a slight increase in Self-Confidence with advancing school years, female children consistently reported lower self-confidence than their male counterparts. According to the literature, at the beginning of primary school, children’s attitudes toward various subjects tend to be generally favorable (Eccles et al., 1993; Jacobs et al., 2002). However, these attitudes typically decline over time (Adelson and McCoach, 2011; Alemany-Arrebola et al., 2025; Dowker et al., 2019; Evans and Field, 2020; Gottfried et al., 2007; House, 2006; Ing and Nylund-Gibson, 2017; Jacobs et al., 2002; Upadyaya and Eccles, 2014; Wen and Dubé, 2022), as academic demands increase, reflecting a broader decrease in academic motivation (Jacobs et al., 2002; Wigfield and Eccles, 2000). Coherently with the Early Math Achievement-Attitude model (Levine and Pantoja, 2021), the decline in children’s mathematical attitudes may be linked to a decrease in academic performance. The increased ability to make social comparisons and a heightened awareness of teachers’ evaluations can also play a significant role in the decline of attitudes toward mathematics (Aunola et al., 2002; Eccles et al., 1993). Some studies have highlighted gender differences in the trajectories of mathematical attitudes, with girls’ interest in mathematics declining as early as primary school, while boys’ interest generally remains higher (Bouffard et al., 2003). Social and cultural factors may significantly influence gender differences in the developmental trajectories of motivation (Eccles and Wigfield, 2020), with gender stereotypes and the expectations of key socializers playing a significant role. Future research could further explore the factors behind these gender differences in the development of mathematical attitudes. In our study, the general decline in positive attitudes toward mathematics, alongside a slight improvement in self-confidence, suggests a complex relationship between self-confidence and overall attitudes toward mathematics. While children reported more self-confidence as they progressed through grades, they seemed to experience a decrease in the perceived value of mathematics, enjoyment, and motivation to continue studying it. Although cross-sectional studies have generally found a positive relationship between ability-related beliefs and value beliefs, as proposed by EVT, longitudinal studies have produced more mixed findings (Arens et al., 2019; Chen et al., 2023; Chung and Kim, 2022; Feng et al., 2024; Lee and Seo, 2021; Pinxten et al., 2014). These relationships may differ depending on factors such as regional culture and the specific facets of attitudes being examined (Arens et al., 2019; Lee and Seo, 2021). Expectancy-value theory underscores the importance of longitudinal research to explore the interrelations between ability-related beliefs and value beliefs throughout different stages of students’ development (Eccles and Wigfield, 2020). A tool that captures multiple dimensions of attitudes toward mathematics enables the examination of individual components’ trajectories over time and facilitates the design of targeted interventions to foster students’ motivation and promote favorable attitudes toward mathematics. Additionally, considering the early decline in favorable attitudes toward mathematics, interventions should prioritize young students to prevent the onset of negative trajectories.

This study further contributes to the literature on the relationship between attitudes toward mathematics, mathematics anxiety, and mathematical competence. Specifically, we found weak but positive correlations with mathematical competence. This finding aligns with the existing literature on the relationship between attitudes toward mathematics and mathematical competence in children, a field in which research has yielded mixed results, with some studies reporting no or weak associations between attitudes and competence (Cain-Caston, 1993; Krinzinger et al., 2009). This is also consistent with previous research suggesting that the relationship between attitudes toward mathematics and mathematical competence strengthens with age (Dowker et al., 2019; Wigfield and Cambria, 2010). Indeed, as children progress through grades, they experience a series of successes and failures that may shape their attitudes and self-confidence in mathematics (Eccles et al., 1993), suggesting that the effects of attitudes on mathematical competence may become more evident over time. These modest correlations may also reflect the fact that mathematical competence is a complex construct influenced by multiple factors, with attitudes toward mathematics representing only one contributing component. Future research should consider additional factors not addressed in the present study, such as general cognitive abilities, executive functions, learning strategies, home environment, and instructional methods. Consistent with previous findings, we also confirmed a negative correlation between each dimension of attitudes toward mathematics and mathematics anxiety (Ashcraft and Krause, 2007; Hembree, 1990).

The first mediation analysis showed that attitudes toward mathematics are associated with mathematical competence both directly and indirectly through mathematics anxiety. However, the only attitude that seemed to have both a direct and indirect effect on mathematical competence through mathematics anxiety was self-confidence. Once mathematics anxiety was included as a mediator, the relationships between Value, Enjoyment, Motivation, and mathematical competence became non-significant, with Self-Confidence remaining the only significant explanatory variable. This highlights the importance of addressing both self-confidence and mathematics anxiety in interventions aimed at supporting students’ learning, as perceived ability and math anxiety are key factors for success in mathematics. Studies on the relationship between attitudes and mathematical competence have yielded conflicting results regarding the extent to which attitudes predict mathematical competence and which attitudes serve as the strongest predictors. Individual components of attitudes toward mathematics may have distinct effects on academic performance, with self-confidence potentially playing a particularly influential role (Abed and Alkhateeb, 2000; Pinxten et al., 2014). Within the framework of EVT, one of the most significant findings is that competence-related beliefs are strongly associated with performance and achievement, whereas task values are primarily associated with engagement and long-term educational trajectories (Arens et al., 2011; Rosenzweig et al., 2022; Wang, 2012). Regarding specific value components, attainment value and utility value appear to be particularly influential in shaping long-term academic participation, whereas intrinsic value is more strongly linked to daily engagement in academic activities. Additionally, perceived cost has been associated with avoidance behaviors (Rosenzweig et al., 2022).

Given the reciprocal nature of the relationships among these constructs, a reverse mediation model was tested to investigate whether mathematical competence is associated with attitudes toward mathematics both directly and indirectly through mathematics anxiety. The results provided evidence for a significant indirect effect, suggesting that students who demonstrate higher mathematical competence tend to experience lower math anxiety, which in turn is associated with more favorable attitudes toward mathematics. This finding underscores the central role of anxiety as a psychological mechanism linking actual mathematical competence to students’ motivational beliefs and emotional responses toward mathematics. Importantly, math competence showed indirect effects on all attitude dimensions, whereas the direct effects on Value and Motivation were non-significant. These findings reinforce the idea that reducing students’ anxiety may be just as important as fostering competence itself to improve motivational outcomes. Notably, among the different dimensions of attitudes toward mathematics, Self-Confidence emerged as the most strongly associated with both math competence and math anxiety. This suggests that students’ perceptions of their own mathematical ability are particularly sensitive to variations in both actual mathematical competence and emotional responses. The result aligns with the Early Math Achievement-Attitude (EMAA) model (Levine and Pantoja, 2021), which describes a developmental shift in the directionality of the relationship between mathematical competence and attitudes. While early mathematical competence is proposed to shape attitudes in young children, the model suggests that, with age, this relationship becomes increasingly bidirectional, as attitudes begin to influence achievement outcomes. In this context, attitudes, − particularly self-confidence - not only reflect the influence of prior mathematical competence via reduced anxiety but may also reinforce future performance, thereby contributing to a reciprocal relationship in which competence and attitudes mutually influence one another over time.

Taken together, these results not only contribute to literature on the relationship between attitudes toward mathematics, mathematics anxiety, and mathematical competence but also highlight the need for targeted educational interventions. Specifically, given its centrality in both direct and indirect pathways, our data suggests that enhancing student’ self-confidence is crucial for improving mathematical competence. Therefore, we propose that educational interventions should prioritize strengthening mathematical self-confidence, as this would enable students to approach mathematics with greater assurance and improve their mathematical competence (Levine and Pantoja, 2021). At the same time, it is important to recognize that attitudes toward mathematics are closely interconnected, with changes in one component potentially influencing the others (Cho and Hwang, 2019). Moreover, given the early decline in favorable attitudes observed in our data, such interventions may be most effective when introduced early in formal education (Cvencek et al., 2011; Jacobs et al., 2002; Steffens et al., 2010).

Teachers can foster self-confidence by creating supportive learning environments and adjusting task difficulty according to students’ abilities, ensuring that all students experience success (Hwang and Son, 2021). Furthermore, considering that mathematics is often perceived as an abstract and challenging subject (Hoyles, 2018; Utami and Hwang, 2022), instructional methods that make abstract concepts more tangible are essential. Strategies such as using manipulatives, real-world applications, and active student participation have been shown to enhance both engagement and confidence (Irvine, 2020; Siller and Ahmad, 2024). A particularly promising approach involves increasing the perceived utilitarian value of the subject (e.g., Hwang and Son, 2021; Lazowski and Hulleman, 2016; Yeager and Walton, 2011). Expectancy-value theory posits that students who recognize the relevance of mathematics to their personal goals attribute a higher utilitarian value to math, which fosters persistence in mathematical activities and ultimately enhances self-confidence and success in mathematics (Rosenzweig and Wigfield, 2016; Yeager and Walton, 2011). Collaborative learning, where students engage in collective problem-solving and knowledge construction, further supports these outcomes, leading to improved academic success and more favorable attitudes toward mathematics among primary school students (Ahmad and Dogar, 2023; Gillies, 2019; Huang et al., 2012; Johnson and Johnson, 2018; Kim et al., 2022; Mathias et al., 2023; Nazari, 2023; Obafemi et al., 2023). Empirical evidence indicates that collaborative learning enhances students’ understanding of mathematical concepts (Hadwin et al., 2017; Malmberg et al., 2017; Slavin, 2015), increases engagement, reduces math anxiety, and bolsters confidence in mathematical abilities (Dolmans, 2019; Gillies, 2016; Gillies et al., 2023; Huang et al., 2012; Johnson and Johnson, 2018; Kalogeropoulos et al., 2023; Khun-Inkeeree et al., 2017; Muis et al., 2018). Teachers can enhance lesson engagement by incorporating game-based learning (Beyhan and Tural, 2007; Elçi, 2017; Vankúš, 2021). Educational games and math training software have been found to increase students’ motivation and their active participation in lessons (Deng et al., 2020; Gök, 2020; Moon and Ke, 2020). Game-based learning also provides opportunities for success in a non-threatening environment, thereby enhancing self-confidence (Balt et al., 2022). Combining multiple intervention strategies may further amplify positive outcomes. For instance, Lee et al. (2021) demonstrated the effectiveness of an intervention aimed at fostering a growth mindset and reducing gender stereotypes in primary school children, leading to improved perceptions of mathematical competence, perseverance, and achievement.

School administrations can enhance mathematics instruction by providing educational resources, such as technological devices, and implementing professional development programs that equip teachers with diversified instructional strategies to make learning more engaging (Hwang and Son, 2021). Encouraging positive math interactions with key socializers, such as parents, also strengthens children’s math self-concept (Levine and Pantoja, 2021). To support this, educational organizations should inform parents about educational applications, which, despite their academic benefits, are not always perceived positively (Vaiopoulou et al., 2021). Additionally, integrating strategies for fostering mathematical self-confidence into teacher training is crucial. To create a supportive learning environment, teachers must be trained to identify, assess, and manage low self-confidence and high math anxiety in the classroom. Given the impact of self-confidence and math anxiety on students’ competence, incorporating targeted training into teacher education would not only enhance teachers’ ability to assist students facing confidence-related challenges but also play a key role in supporting students’ sustained engagement and success in mathematics. Promoting gender equity in mathematics education is another key priority (Mukagiahana et al., 2024; Nimely and Nyamu, 2023). Evidence indicates that teacher training initiatives designed to reduce implicit biases in mathematics, particularly those related to gender, have proven effective (Feierabend et al., 2024; Kollmayer et al., 2016). To cultivate inclusive learning environments that promote equitable participation in mathematics, teachers should actively challenge traditional gender roles and ensure that all students are given equal opportunities to engage with mathematical content, regardless of gender (Nimely and Nyamu, 2023).

However, it is important to acknowledge that the successful implementation of these recommendations may present several challenges. A significant challenge lies in ensuring that interventions are effectively tailored to the unique needs of diverse educational contexts. For example, school curricula and educational priorities may vary, meaning that strategies designed to enhance mathematics instruction might not always align with the specific needs of every school or classroom. The diversity of students’ learning styles and varying levels of teacher preparedness can also influence the success of these interventions. Therefore, addressing these challenges and considering the context in which these interventions will be implemented is crucial for maximizing their impact. Future research could examine these barriers and provide further insight into how interventions can be adapted to overcome such challenges, ensuring their applicability and success across various educational settings. Another potential barrier is institutional constraints, such as limited funding or resources for technological devices and professional development programs. At the classroom level, teachers often face significant limitations in adapting curricula or lesson plans due to constraints on time and resources. Therefore, achieving broader educational change is a complex challenge that requires integrating school reform efforts with evidence-based practices (Rosenzweig et al., 2022).

### 4.1 Limitations and future directions

This study presents several limitations. First, although the sample size was relatively large, participants were recruited exclusively from three schools located in central Italy. This restricted sampling frame may limit the extent to which the findings can be generalized to children from different geographical, cultural, or socio-economic contexts. Attitudes toward mathematics can be shaped by a variety of cultural and contextual factors, such as societal expectations surrounding academic achievement, warranting further cross-cultural validation. Future research should therefore consider including participants from a broader range of educational and socio-cultural settings, to test the cross-cultural validity of the ATMI-SF-C and verify whether its psychometric properties remain stable across diverse contexts. Adapting the ATMI-SF-C for geographically and culturally diverse samples may help to explore the influence of cultural factors on attitudes towards mathematics and assess the instrument’s applicability across different educational frameworks, enhancing the generalizability of the findings. While the ATMI-SF-C has demonstrated reliability and validity within the current sample, its performance in different educational settings remains an important avenue for further research. Educational environments can vary significantly in terms of curriculum structure, teaching methodologies, and cultural perceptions of mathematics, all of which may influence how students interpret and respond to the ATMI-SF-C items. For example, research suggests that Asian students are likely to develop unfavorable attitudes toward mathematics due to the high expectations set by their socializers, including teachers and parents (Kung and Lee, 2016; Martin et al., 2020; Papanastasiou, 2000; Uchida and Mori, 2018). Indeed, given the strong emphasis placed on mathematics achievement, Asian parents and teachers often encourage rigorous study and high performance on assessments, which can contribute to the formation of unfavorable attitudes toward math (Hwang and Son, 2021). Future research should examine the psychometric properties of the ATMI-SF-C across diverse educational contexts to assess whether its factor structure, reliability, and validity remain consistent across different settings. Conducting such cross-contextual analyses would enhance confidence in the instrument’s applicability and provide insights into how educational and cultural factors shape attitudes toward mathematics.

In addition, participants in the present study were nested within schools. Future studies, particularly those including participants recruited from broader and more diverse socio-cultural contexts, could test the model by accounting for the clustering effect of the school variable. Additionally, the reliance on self-reported instruments, although adapted for this age group using simple items and a visual response scale, may have introduced potential biases. Children may have difficulty describing their psychological condition using graduated scales (Varni et al., 2001), due to limited cognitive ability, memory skills, and attention span (Eddy et al., 2011). Research indicates that younger children may be more susceptible to response biases, such as acquiescence, social desirability (Logan et al., 2008) or extreme responding (Chambers and Johnston, 2002; Davis et al., 2007) when rating emotional states, and that the ability to provide reliable responses improves with age (Rebok et al., 2001). Moreover, exclusive reliance on self-report measures may have inflated associations due to common methods effects. Therefore, the validity of the ATMI-SF-C should be further examined through mixed models that combine self-reported measures with direct observations, teacher ratings or performance-based tasks, to strengthen the validity of the results and reduce potential biases arising from using a single method in the same session. Validating self-reported data through comparison with teacher assessments could also strengthen the robustness of findings. Multi-informant approaches, which integrate perspectives from key figures in the child’s life (e.g., teachers and parents), have been recommended to counterbalance the limitations of individual self-report measures (e.g., De Los Reyes et al., 2015). Moreover, the application of the validity-index approach can further assess whether self-reports yield data of comparable psychometric quality to those provided by parents or other informants (Conijn et al., 2020). By incorporating these strategies, future studies could enhance the validity of the ATMI-SF-C and mitigate the impact of common method bias.

Given the correlational nature of the study, the stability of the tool over time was not assessed. Furthermore, the cross-sectional design of the study did not allow for the capture of developmental trajectories or the determination of causal relationships between variables. While trend analyses provide valuable insights, longitudinal studies are needed to explore the trajectory of attitudes over time and provide insights into the long-term development of mathematical attitudes. In addition, the temporal stability of the ATMI-SF-C has not yet been evaluated, and future research should assess its reliability when administered repeatedly to ensure it provides consistent measurements, which are essential for educational monitoring. Future studies should also examine the longitudinal stability of the measure and its sensitivity to intervention effects. Additionally, longitudinal research could provide valuable insights into the predictive validity of the ATMI-SF-C scores in relation to other variables and educational outcomes. The ATMI-SF-C appears to be a valuable instrument for capturing the dynamic and reciprocal relationship between attitudes toward mathematics and mathematical competence. From an educational perspective, it is essential to recognize the circular nature of these relationships, since students’ self-perceptions, emotional responses, and actual mathematical competence interact over time in ways that may either reinforce academic growth or perpetuate disengagement and underachievement. Therefore, supporting students’ success in mathematics requires not only the development of cognitive competence but also ongoing efforts to strengthen motivational beliefs and reduce affective barriers. To more accurately understand the causal nature of these associations and how their reciprocal influences evolve, future research should employ longitudinal models capable of capturing developmental changes and directionality in these relationships.

Lastly, the study did not investigate the relationships between the different dimensions of attitudes towards mathematics and other related constructs, such as general or academic self-efficacy. It also focused solely on the relationship between attitude and basic mathematical tasks, including number representation and mathematical calculation. Therefore, the identified relationships may not fully capture the connection between attitudes towards mathematics and a broader range of mathematical activities. Additionally, it is important to investigate the relationship between attitudes towards mathematics and academic assessments, as these have been shown to be among the best longitudinal predictors of future mathematical competence in primary education (Arens et al., 2017). Unlike tools that assess mathematical competence, academic evaluations also capture other critical aspects of student learning, including engagement, motivation, interest, and classroom behavior, all of which are all relevant for future development and success (Master and Meltzoff, 2020; McMillan et al., 2002).

## 5 Conclusion

Despite these limitations, this study provides evidence of the good psychometric properties of the ATMI-SF-C in a sample of primary school children, supporting its use as a multidimensional tool for measuring attitudes toward mathematics in this age group. The availability of a reliable tool for assessing these attitudes opens new research opportunities in primary education. Exploring the relationship between parents’, teachers’, and children’s attitudes towards mathematics, along with contextual variables such as gender stereotypes in mathematics, could deepen our understanding of how these attitudes develop. The multidimensional nature of the ATMI-SF-C is particularly advantageous, as it allows for the independent examination of the various components of attitudes toward mathematics. Future research could explore how individual components of attitudes towards mathematics develop throughout schooling and their influence on educational and career choices. Furthermore, future studies could focus on developing proxy measures of attitudes towards mathematics for parents and teachers. Finally, the ATMI-SF-C represents a valuable tool for educators and policymakers, providing insights that can inform the design of interventions aimed at fostering more favorable attitudes toward mathematics in primary school children. Its brevity also makes it an efficient and easy-to-apply instrument for assessing the effectiveness of such interventions.

## Data Availability

The raw data supporting the conclusions of this article will be made available by the authors, without undue reservation.
