# Supplementary material for: The utility of the Attitudes Toward Mathematics Inventory—Short Form for Children for assessing attitudes toward mathematics in primary school children
Source: Front Psychol. 2026 Jan 22;17:1659707. doi: 10.3389/fpsyg.2026.1659707 (PMC12872822; doi:10.3389/fpsyg.2026.1659707)
Supplement: Supplementary file 1 [file Table_1.DOCX]

Supplementary materials

Table S1. *Means (M), standard deviations (SDs), skewness, and kurtosis of the 14 items of the ATMI-SF-C.*

| Item | *M* | *SD* | Skewness | Kurtosis |
| --- | --- | --- | --- | --- |
| 1 | 4.39 | .88 | -1.56 | 2.42 |
| 2 | 4.04 | 1.00 | -.89 | .24 |
| 3 | 3.74 | 1.14 | -.62 | -.35 |
| 4 | 3.75 | 1.22 | -.69 | -.50 |
| 5 | 3.81 | 1.32 | -.79 | -.58 |
| 6 | 3.99 | 1.27 | -1.03 | -.13 |
| 7 | 3.89 | 1.20 | -.84 | -.24 |
| 8 | 3.66 | 1.36 | -.64 | -.83 |
| 9 | 3.53 | 1.40 | -.48 | -1.04 |
| 10 | 3.81 | 1.33 | -.84 | -.48 |
| 11 | 3.76 | 1.23 | -.70 | -.48 |
| 12 | 2.86 | 1.45 | .11 | -1.31 |
| 13 | 2.60 | 1.37 | .34 | -1.07 |
| 14 | 3.42 | 1.42 | -.40 | -1.13 |

*Note.* ATMI-SF-C = *Attitudes Toward Mathematics Inventory - Short Form - Children.* Likert scale ranges from 1 = *I don’t agree much* to 5 = *I very much agree. N* = 798.
